# Application of acidic deep eutectic solvents in green extraction of 5-hydroxymethylfurfural

**DOI:** 10.1038/s41598-022-16823-x

**Published:** 2022-07-30

**Authors:** Sabah Karimi, Hemayat Shekaari

**Affiliations:** Department of Physical Chemistry, Faculty of Chemistry, University of Tabriz, Tabriz, Iran

**Keywords:** Chemical engineering, Thermodynamics

## Abstract

The extraction of the 5-hydroxymethylfurfural (5-HMF), as building block for many applications, from aqueous solutions has been became an indispensable challenge. Consequently, this study investigated the extraction ability of acidic deep eutectic solvent-based aqueous two-phase system (ATPS), choline chloride as hydrogen bond acceptor (HBA) and lactic acid, oxalic acid and citric acid as hydrogen bond donor (HBD), countering polypropylene glycol 400 at *T* = 298.15 K. Two semi-empirical Zafarani-Moattar et al. and Merchuk equations were used to fit the measured binodal data. Further, the NRTL and UNIQUAC models were used for correlating of tie-line data. The consistency of the experimental tie-line data was determined by utilizing the Bachman-Brown and Hand correlations. Also, the performance of these ATPSs to partitioning of 5-HMF, were investigated by calculation of extraction efficiencies, *EE*% and partition coefficients, *K*. This strategy indicates DES-based ATPSs have the acceptable extraction efficiency of the 5-HMF in a single-step extraction. A greenness assessment tool, Analytical GREEnness metric (AGREE), was tested to evaluate of greenness of analytical protocol.

## Introduction

From the viewpoints of environmental protection, energy security and economic development, it is extremely urgent to exploit the proper renewable resources to decrease the excessive dependence on the non-renewable fossil resources^1^. Biomass as a renewable energy source for energy and chemicals has great potential value and are widely recognized^2^. 5-hydroxymethylfurfural (5-HMF) is a key platform chemical being derived from the carbohydrates dehydration and can be further converted to many valuable fuels, and it has the potential of commodities like terephthalic and levulinic acid^3–8^. 5-HMF is made up of a furan ring with two functional groups: one alcohol group and one aldehyde group, therefore, it has acceptable solubility in both organic solvents like ethyl acetate and polar solvents like water, therefore, the extraction of 5-HMF has been become an utmost important challenge^9–11^.

Up to now, traditional separation and extraction methods including various forms of adsorption, and physical adsorption, column chromatography and water–oil two-phase extraction are applied^12^. The extraction operations using these methods are low product yield and complicated. The 5-HMF rich stream was purified downstream via liquid–liquid extraction using dichloromethane as a solvent and boasted a 98% 5-HMF recovery potential. This process requires additional separation steps in the form of three flash tanks where vacuum evaporation recovers almost 96.5% of pure 5-HMF. The flash tanks operated at temperatures lower than 80 °C and under vacuum to prevent 5-HMF degradation^13^. Numerous biphasic-solvent systems with different homogenous and heterogeneous catalysts have been extensively studied over the years, especially after Rom´ an-Leshkov presented the batch process set-up for the production of 5-HMF from fructose with simulated counter-current extraction and evaporation steps^14^. Several biphasic solvent systems, including systems relying on the “salting out” effect from NaCl or HCl, have been investigated with the use of zeolites with varying success. Some homogeneous acid catalysts can be corrosive and require more cautious handling as heterogeneous catalysts can be expensive and uneconomical^15^. In 1896, Martinus Willem Beijerinck accidently found the aqueous two phase system (ATPS) while mixing an aqueous solution of gelatin and starch^16^. However, Per-Åke Albertsson discovered the real application^17^. An ATPS, which can provide the low operational cost isolation and non-toxic process besides large-scale and fast purification, has become an ideal technique for extraction process^18^.

Phase separation in aqueous solutions containing polymer like polyethylene glycol or polypropylene glycol in presence of salts is a very common phenomenon, which this effect depending on both concentration and type of salt^19^.

Extraction of 5-HMF, as a foundation of modern biofuel synthesis, has recently been under intensive study by a multitude of research groups^20^. Recently investigators have studied the effects of solvent and salt types on thermodynamics of 5-HMF extraction. However, the use of salt in the extraction process in aqueous phase can enhance the partitioning of 5-HMF into organic phase. Different salts made up of various cations and anions were investigated, such as; KCl, NaCl, K_2_SO_4_ and Na_2_SO_4_, and different solvents like alcohol, ketone have been utilized on the partitioning of 5-HMF in two phase systems^21^. To prediction the LLE systems the COSMO-SAC model, and to correlation of the experimental LLE systems including 5-HMF, the NRTL and UNIQUAC models have been used^22^. Despite their attempts toward the extraction of 5-HMF, it is more urgent to find an efficient and green extraction method.

Ionic liquid (IL) is a salt in the liquid state in which the ions are poorly coordinated and results in these solvents being liquid below 100 °C. Most recently, ILs based ATPSs are being developed^23–25^. Nevertheless, compared with organic solvents, they are more costly and difficult to synthesis, and their eco-friendliness has been challenged.

Deep eutectic solvents (DESs), as a new generation of ILs, have the shared physico-chemical properties with ILs, but their preparation is much cheaper, lower cost and simpler which formed by mixing ammonium salt as hydrogen bond acceptor (HBA), and aprotic organic compound as hydrogen bond donor (HBD)^26^. DESs were widely used in the extraction field^27,28^. The HBA (quaternary ammonium salts) shows prominent potential to the salting-out, as well as, the polarity of HBD groups influence on phase-forming ability. The main objective of this study is to find out the using DESs as extraction agent in room temperature. The term Green Analytical Chemistry (GAC) emerged in 2000 in an attempt to reduce the harmful effects of analytical practices on human beings and the environment. The principles of GAC are represented in the word SIGNIFICANCE: S—selection of a straight analytical technique, I—integration of analytical operations and processes, G—generation of minimum waste, N—never wasting energy, I—implementation of method automation, F—favoring fewer toxic reagents, I—increasing operator safety, C—carrying out in situ measurements, A—avoidance of derivatization, N—noting the use of a smaller number of samples, C—choice of multi-analytical methods, E—elimination of toxic reagents. Recently, AGREE, a novel downloadable greenness assessment software, has been proposed by Pena-Pereira and his fellows in June 2020. It relies on the twelve basics of GAC, SIGNIFICANCE, as mentioned earlier in the introduction^29,30^.

Recently, our research team has investigated the effect of DESs on thermodynamic properties of 5-HMF^31^. Continuing in this line of research, this study is focused on the effect of DESs (ChCl:lac, ChCl;ox and ChCl:cit in molar ratios 1:1) on 5-HMF extraction and experimental binodal data and tie-lines determination of (DES + PPG400 + water) at T = 298.15 K and under pressure (85 kPa). Furthermore, the NRTL and UNIQUAC, activity coefficient models, are widely applied for describing phase equilibrium data. The experimental binodals were correlated with two empirical equations involving Zafarani-Moattar and coworkers and Merchuk. The consistency of the measured LLE data were tested using the Hand and Bachman–Brown. Furthermore, the partition coefficients of 5-HMF were correlated using the Diamond-Hsu model. Then, AGREE used as a greenness assessment tool for evaluation of greenness of analytical protocol.

## Experimental section

### Materials

All data corresponding to the chemicals used in this study have been shown in Table 1. All materials were used without further purification. Choline chloride was purchased from Fluka company and the deionized water was utilized in preparation of solutions. 5-HMF, lactic acid, oxalic acid and citric acid were purchased from Sigma Aldrich company as well as, silver nitrate, potassium chromate, sulfuric acid and sodium hydroxide were purchased from Merck company.

**Table 1:** All data corresponding to used materials in this work.

Material	Abbreviation	CAS no	Purity
Choline chloride	ChCl	67-48-1	≥ 0.99
PPG400	PPG	25322-69-4	> 0.99
Lactic acid	lac	79-33-4	> 0.85
Oxalic acid	ox	6153-56-6	≥ 0.99
Citric acid	cit	77-92-9	≥ 0.99
Silver nitrate	–	7761-88-8	≥ 0.99
Potassium chromate	–	7789-00-6	≥ 0.99
Sulfuric acid	–	7664-93-9	≥ 0.95
Sodium hydroxide	–	1310-73-2	≥ 0.99

### DES preparation

The hydrogen bond acceptor (HBA:choline chloride) is combined with hydrogen bond donor (HBD:carboxylic acids) with the same molar ratio of 1:1. The HBA and HBD were magnetically stirred and heated at 368.15 K until the mixtures became a homogeneous colorless liquid.

### Liquid–liquid equilibrium measurements

For every systems, the cloud-point measurements method was applied to obtain the binodal curves^32,33^:

(I) The aqueous solution of DES was added drop by drop to a mixture of aqueous solution of PPG400 with certain concentration. When the appearance of the system changes from clear to turbidity, one point of boundary composition is determined, and so on, to obtain the boundary desired.
(II) Water was added drop by drop to mixture of obtained solution in section I. When all the crystals in the solution were just dissolved completely and the solution became clear, one point on the solubility curve is determined, and obtain the binodal curve.

### Experimental procedure

The Carl Fischer (Metrohm 751 GPD) method was used to determinate of water content of choline chloride, carboxylic acids and obtained DESs, which it was less than 0.1% (w/w). The mixture composition was determined using an analytical balance (Shimadzu, 321-34,553, Shimadzu Co. Japan) with precision of 1 × 10^–5^ g with a precision of ± 1 × 10^–4^ g. To ensure the reproducibility and accuracy of the obtained data, the cloud point method for each system, were performed in three replicates. A lamp was also located near the measuring glassy cell for better visualization of the exact DESs mass fractions related to binodal curves. These binodal curves for aqueous solutions of ChCl:lac 70 wt% and PPG400 80 wt%, ChCl:ox 70 wt% and PPG400 80 wt%, ChCl:cit 60 wt% and PPG400 80 wt%, were determined.

For each tie-line, a mixture point in the two-phasic region of each ternary system was gravimetrically prepared and centrifuged to phase separation and placed in water bath at 298.15 K to reach equilibrium for a minimum of 6 h. After the equilibration step, the top and bottom phases were carefully separated and weighed with a precision of ± 10^–5^ g. The compositions of coexisting phases were analytically determined.

Mohr method^34^, known as argentometric method, is one of the significant methods for determination of chloride in water. Choline chloride is a quaternary ammonium salt with choline cation and chloride anion. This method determines the chloride ion concentration of a solution by titration with silver nitrate using potassium chromate as indicator.

There are some common chemical indicators that are utilized with argentometric titrations: (I) The chromate ion, ${\text{CrO}}_{4}^{2 - }$ (the Mohr method); (II) adsorption indicators such as fluorescein (the Fajans method); (III) The ferric ion, Fe^3+^ (the Volhard method)^35^.

Mohr indicator reaction is based on the following reactions:

$$ {\text{Cl}}^{ - } + {\text{AgNO}}_{3} \to {\text{AgCl}} + {\text{NO}}_{3}^{ - } $$

$$ 2{\text{Ag}}^{ + } + {\text{CrO}}_{4}^{2 - } \to {\text{Ag}}_{2} {\text{CrO}}_{4} $$

The concentration of titrant rises sharply near the equivalence point, and the solubility of ${\text{Ag}}_{2} {\text{CrO}}_{4}$ is exceeded. The appearance of red precipitate marks the endpoint.

1 $$ ppm\left( {\frac{{{\text{mg}}}}{{\text{l}}}\;as\;{\text{Cl}}^{ - } } \right) = \frac{(A - B) \times N \times 35{,}460}{V} $$

where A and B are the volumes of ${\text{AgNO}}_{3}$ for sample and blank, respectively. The normality of ${\text{AgNO}}_{3}$ is N, and V is the volume of water sample taken.

Commercial racemic, d- or l-lactic acid are usually aqueous solutions 82–95 wt%. Also, lactic acid presents dimer, trimer and oligomer states in equilibrium with monomeric lactic acid. The back-titration is an utmost important method to quantify the total acidity of lactic acid. Back-titration is applied when the reaction is too slow or no suitable sensor is available for a practical direct titration^36^. For this purpose, first, at the boiling temperature, lactic acid reacted with sodium hydroxide using phenolphthalein indicator, and then using a standard solution of sulfuric acid, the excess alkali was back titrate. The samples containing 1 gr from each phase were weighed and dissolved with 25.0 mL of deionized water, combined with 20.0 mL of 0.1 M sodium hydroxide standard solution, boiled for 10 min to hydrolyze oligomers of lactic acid completely. Eventually, back titration was applied to determine the excess alkali with the sulfuric acid standard solution of 0.05 M using phenolphthalein as a visual indicator. The total acidity was calculated as following equation^37^:

2 $$ {\text{Total acidity }} = \frac{{C_{{{\text{NaOH}}}} \times (V_{Sample} - V_{Blank} ) \times 90.08}}{m \times 1000} \times 100\% $$

where $m$ is weight of the sample and $C_{{{\text{NaOH}}}}$ is the ${\text{NaOH}}$ standard solution concentration.

To quantify the concentration of PPG400 in each phase, The refractometer (ATAGO DR-A_1_, Japan) with a preision of 0.0001 was used as presented by Cheluget et al.^38^. In refractive index measurements, the estimated uncertainty is 0.0002. According to this method, there is the following relation between the mass fractions of PPG400, *w*_p_, choline chloride, *w*_c_ and carboxylic acids, *w*_*a*_ and refractive index, *n*_D_:

3 $$ n_{D} = n_{0} + a_{p} w_{p} + a_{c} w_{c} + a_{a} w_{a} $$

The values of constants *a*_p_, *a*_c_ and *a*_a_ corresponding to PPG400, choline chloride and carboxylic acids, respectively were calculated from the linear calibration plots of the refractive index of the solution and $n_{0}$ = 1.3325 is the refractive index of pure water at *T* = 298.15 K. The coefficient of determination values *R*^2^ and values of these constants together are reported in Table S1. In each phase, the sum of mass fractions is equal to unity ($w_{w} + w_{p} + w_{c} + w_{a} = 1$). To determine the concentrations of both of choline chloride and polymer, two equations with tow unknown weight fraction ($w_{p}$ and $w_{c}$) were used. The uncertainty of the mass fraction of each of the ATPS components was about 0.008. Since, the Eq. (3) is only valid for dilute solutions, the samples, before refractive index measurements, were diluted in the mass fraction range (C *Range(w/w)*) as given in Table S1.

### Partition coefficient

To evaluate extraction efficiency and determine the performance of the considered ATPSs for 5-HMF partitioning, based on the phase diagrams determined for {PPG400 + DES (ChCl: carboxylic acids) + H_2_O} systems, the mixture compositions with similar overall compositions used to get tie-lines reported in Table S2. After stirring these mixtures for 25 min and observing the phase separation, the mixtures were placed in a water bath. Then 0.002 mass fractions of 5-HMF was added to 1 mL of each phase. The samples were centrifuged at a speed of 3000 rpm for 20 min and maintained in water bath for 24 h to ensure complete separation of the phases and reach equilibrium. Finally, after separation of phases, the 5-HMF in both phases was measured by UV spectroscopy (Model: SPECORD 40-Series Analytik Jena AG- Germany) at the maximum wavelength of 286 nm.

### Analytical GREEnness metric (AGREE)

In AGREE, the final score is a fraction of unity, from zero to one. The automatically resultant pictogram is divided into twelve sections with flexibility for controlling their width according to their importance. Each section has a specific color range from deep green (= 1) to deep red (= 0). The overall score appears in the middle of the circular pictogram. The AGREE too was downloaded from a specific link provided in an AGREE article on November 15, 2020^39,40^.

## Results and discussion

DES-based LLE is an important separation method in research and chemical analysis. As a commercial process, it is frequently used in the chemical and mining industries and in the downstream recovery of fermentation products (antibiotics, amino acids, antioxidants)^41^.

### Phase diagram

The phase diagram is a graphical representation of the physical states of a substance under different conditions and provides information about concentration of phase forming components in the top and bottom phases. Thus, the binodal curve is important for researchers to understand the liquid–liquid equilibrium. For all phase diagrams, the monophasic region is localized below the biphasic region in the binodal curve^28^.

**Figure 1:**
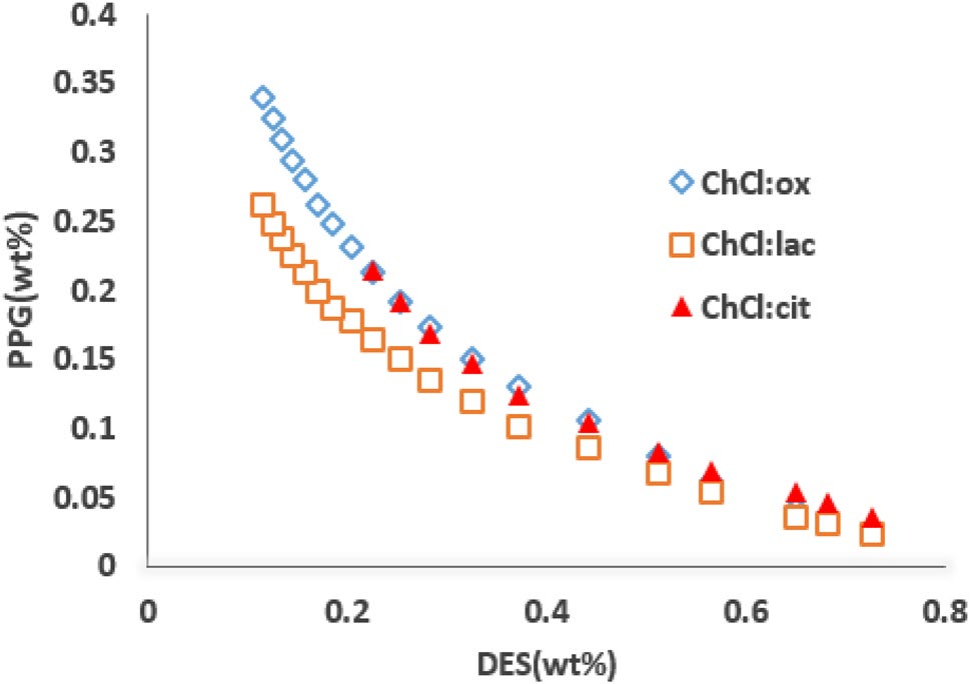
Experimental binodal data of ATPS composed of PPG400, mixtures of DESs and water at 298.15 K.

Three kinds of mono, di and tri-carboxylic acids, namely, lactic, oxalic and citric acids as the HBD, and choline chloride as the HBA, at the same mole composition ratio (1:1), were used to form of DESs. The phase diagrams of some ATPSs composed of DES + PPG400 + H_2_O were obtained by the cloud point titration method at room temperature which depicted in Fig. 1. As shown, the binodal area for citric acid is less than lactic and oxalic acid therefore, it can be seen that the phase forming ability of the DESs fall off in the order: ChCl:lac ≈ ChCl:ox ˃ ChCl:cit. In the considered ATPSs, the bottom phase corresponds to the aqueous solution enriched in DES, while the top phase is mostly composed of PPG400. There are several complicated factors spawned this difference, such as hydrophilicity factor, density and viscosity of the DESs. The hydrophilicity factor causes DES with higher carboxylic functions required more PPG400.

### Determination of the tie-lines length

The tie-line length (TLL) has unit of %w/w, same as the component concentrations. The TLL, which are listed in Table S3, can be related to the equilibrium phase composition as follows^42^:

4 $$ TLL = \sqrt {\left( {w_{P}^{top} - w_{P}^{bot} } \right)^{2} + \left( {w_{HBA}^{top} - w_{HBA}^{bot} } \right)^{2} + \left( {w_{HBD}^{top} - w_{HBD}^{bot} } \right)^{2} } $$

where $w_{p}$, $w_{HBA}$ and $w_{HBD}$ are equilibrium compositions of PPG400, choline chloride and carboxylic acids, respectively. Superscripts “bot” and “top” designate the PPG400-rich phase (top phase) and DES-rich phase (bottom phase), respectively.

**Figure 2:**
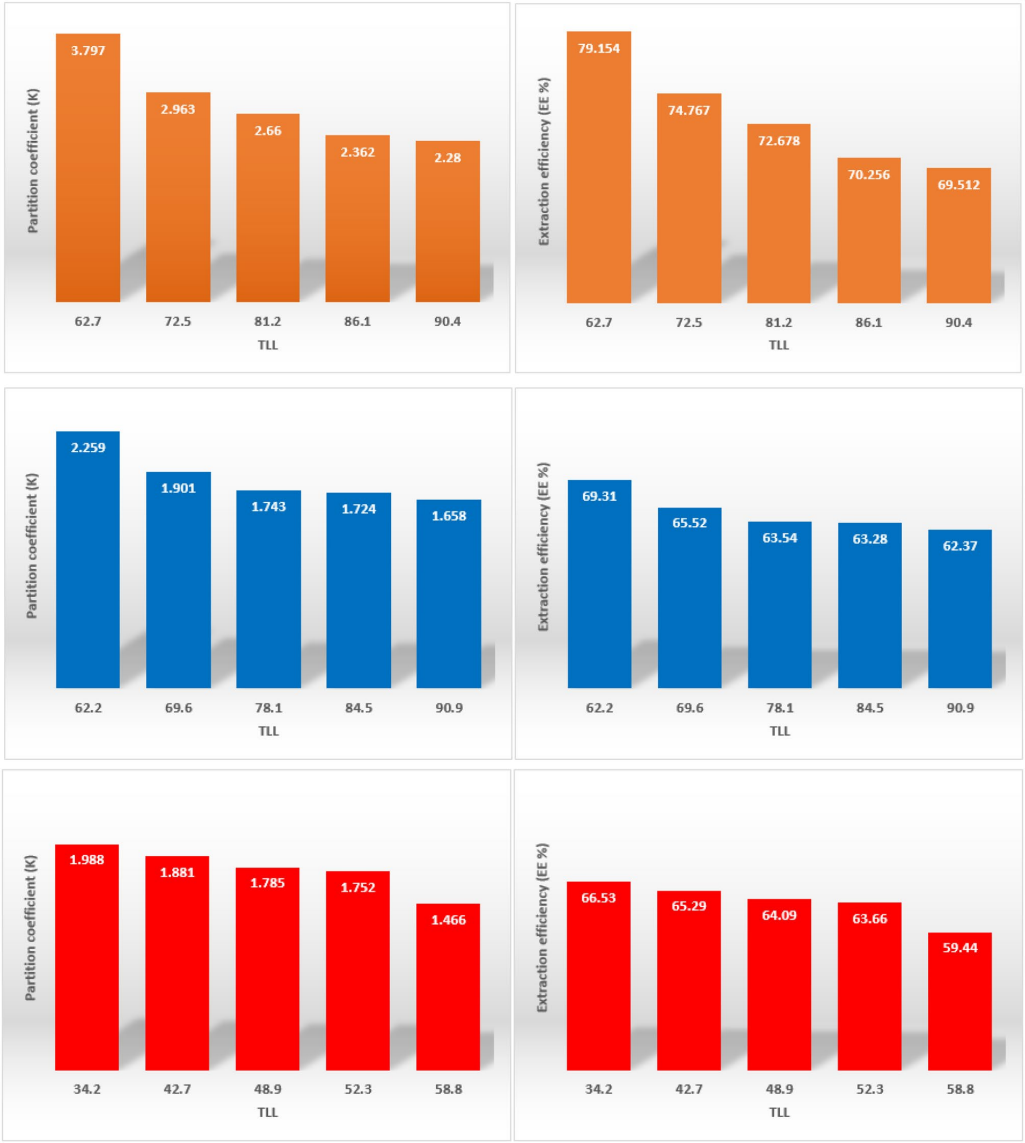
Extraction efficiency and partition coefficient for each studied 5-HMF in ATPSs. ChCl:lac (orange filled rectangle) ChCl:ox (blue filled rectangle) and ChCl:cit (red filled rectangle).

Distribution coefficient (K) (sometimes referred to as partition ratios) is widely used in environmental science to relate the concentration of a chemical solute in one phase to that in a second phase between which equilibrium applies or is approached. The distribution coefficient, measured at equilibrium, reflect the solubility of a compound in both phases, and so is dependent on the solvent system used. Distribution coefficient and extraction efficiencies (EE%) were calculated by the following equations^43^ and the obtained values were listed in Table 2 and shown in Fig. 2.

5 $$ K = \frac{{w_{m}^{top} }}{{w_{m}^{bot} }} $$

6 $$ EE\% = \frac{K}{K + 1} \times 100 $$

**Table 2:** Values of partitioning coefficients, *K*, and extraction efficiency, *EE*%, of 5-HMF for the systems {PPG400 + DES + H_2_O} at 298.15 K and atmospheric pressure (≈ 85 kPa).

Overall composition/wt%		K	EE%
DES	PPG400		
**ChCl:lac**			
14.20	47.69	3.797	79.15
17.15	47.27	2.963	74.76
20.21	47.81	2.660	72.67
22.97	47.69	2.362	70.25
25.82	47.57	1.280	69.51
**ChCl:ox**			
16.52	38.97	2.259	69.31
19.44	38.61	1.901	65.52
22.31	38.82	1.743	63.54
25.64	38.49	1.724	63.28
28.19	38.71	1.658	62.37
**ChCl:cit**			
0.129	57.05	1.988	66.53
0.145	56.98	1.881	65.29
0.161	57.06	1.785	64.09
0.177	57.00	1.752	63.66
0.193	56.99	1.466	59.44

The obtained results reveal that, the 5-HMF spontaneously prefers to migrate to the PPG400-rich phase and the best partitioning with a extraction efficiency of 79% was obtained by the ChCl:lac as DES. The different partitioning behaviors of the three systems can be explained by the hydrophobicity difference between the phases. In terms of their physico-chemical properties, mono, di and tri-carboxylic acids are amphipathic substances, being composed of a hydrophobic hydrocarbon and some hydrophilic carboxylic groups. In terms of their direct effect on the phase formation ability, the more hydrophilic carboxylic groups, the more PPG400 needs and has low efficiency, therefore, the presence of three carboxylic functional groups in citric acid leads to increase hydrophilicity and decrease efficiency.

### Modeling

Once the binodal curve is experimentally obtained, it is important to determine the composition of the upper and bottom phases. To this purpose, in order to avoid lengthy and minute analytical protocols, proposed methods based on least-squares regression methods by Merchuk et al.^44^ (Eq. 7), Zafarani-Moattar et al.^45^ (Eq. 8).

7 $$ w_{p} = a \cdot \exp (bw_{s}^{0.5} - cw_{s}^{3} ) $$

8 $$ w_{p} = \alpha + \beta \ln (w_{s} ) + \gamma w_{s} $$

The concentrations (mass fraction) of PPG400 and DES are denoted as $w_{p}$ and $w_{s}$, respectively, and a, b, c, α, β, γ are the fitted parameters. The adjustable parameters from fitting the binodal data are reported in Table 3. The *sd* reported values in Table 3 illustrated that both equations have an acceptable correlation result.

**Table 3:** Values of parameters of, (*a, b, c*), (*α, β,* γ), and ($v_{{_{P - DES - W} }}^{ * }$, *f*), for {PPG400 + ChCl (HBA) + carboxylic acids (HBD) + H_2_O} systems at 298.15 K.

*DES*	Merchuk			
	a	b	c	sd^a^
ChCl:lac	1.1607	− 2.701	37.1474	0.014
ChCl:ox	1.2507	− 3.2726	15.5132	0.011
ChCl:cit	0.9975	− 1.6409	11.7243	0.0015

	Zafarani-Moattar et al.			
	α	β	γ	sd
ChCl:lac	0.2698	− 0.14	− 1.1726	0.016
ChCl:ox	− 0.0050	0.2069	0.3541	0.014
ChCl:cit	0.504	− 0.0783	− 0.9701	0.001

	Effective excluded volume theory, binodal model			
	$v_{{_{P - DES - W} }}^{ * }$	f	sd	
ChCl:lac	15.1436	2.1866	1.27	
ChCl:ox	15.9824	2.4597	1.76	
ChCl:cit	22.9903	0.6201	1.30	

^a^$sd = \left( {\sum\nolimits_{i = 1}^{N} {(w_{1}^{cal} - w_{1}^{\exp } } )^{2} /N} \right)^{0.5}$ where w1 and N represented mass fraction of PPG400 and number of binodal data, respectively.

The molecular features of a binary solution are described by one parameter which developed by Guan et al., which called the effective excluded volume EEV^46^.

9 $$ ln\left( {v_{P - DES - W}^{ * } \frac{{w_{DES} }}{{M_{DES} }} \, + f_{DES} } \right) + v_{P - DES - W}^{ * } \cdot \frac{{w_{P} }}{{M_{P} }} = \, 0 $$

10 $$ v_{{_{P - DES - W} }}^{ * } = \rho N_{a} \nu_{{_{P - DES - W} }} $$

The $\nu_{{_{p - DES - w} }}$ is the volume of polymer species at constant solution density in presence of DES and $v_{{_{p - DES - w} }}^{ * }$ is effective excluded volume. These fitting parameters, obtained from the correlation of the experimental binodal data along with the corresponding standard deviations, are listed in Table 3.

The non-random two-liquid (NRTL)^47^ and universal quasi-chemical (UNIQUAC)^48^ are the local composition models that used for fitting of tie-line compositions.

The values of the non-randomness parameters were obtained and their optimum value usually set in a fixed value (between 0.01 and 0.4) which in this study it was found to be 0.3. The binary interaction parameters used in the NRTL model for the ternary systems were also determined and are listed in Table 4.

11 $$ \ln \gamma_{i} = \frac{{\sum\nolimits_{j = 1}^{3} {\tau_{ji} G_{ji} x_{j} } }}{{\sum\nolimits_{k = 1}^{3} {G_{ki} x_{k} } }} + \sum\limits_{j = 1}^{3} {\frac{{x_{j} G_{ij} }}{{\sum\nolimits_{k = 1}^{3} {G_{kj} x_{k} } }}\left( {\tau_{ij} - \frac{{\sum\nolimits_{k = 1}^{3} {x_{k} \tau_{kj} G_{kj} } }}{{\sum\nolimits_{k = 1}^{3} {G_{kj} x_{k} } }}} \right)} $$

**Table 4:** Values of parameters of the NRTL and UNIQUAC models for {PPG400 + DES + H_2_O} systems at *T* = 298.15 K.

NRTL						
$\tau_{DES,P}$	$\tau_{P,DES}$	$\tau_{DES,W}$	$\tau_{W,DES}$	$\tau_{W,P}$	$\tau_{P,W}$	$Sd$
**ChCl:lac**						
3.249 × 10^7^	7.862 × 10^3^	1.434 × 10^3^	1.61 × 10^4^	540.239	1.078 × 10^4^	0.005
**ChCl:ox**						
9.578 × 10^3^	5.245 × 10^3^	1.211 × 10^4^	2.044 × 10^4^	1.42 × 10^3^	1.222 × 10^4^	0.012
**ChCl:cit**						
3.707 × 10^5^	7.945 × 10^3^	531.365	2.037 × 10^4^	684.696	8.48 × 10^3^	0.005

UNIQUAC						
$\Delta u_{DES,P}$	$\Delta u_{P,DES}$	$\Delta u_{DES,W}$	$\Delta u_{W,DES}$	$\Delta u_{W,P}$	$\Delta u_{P,W}$	$Sd$
**ChCl:lac**						
1.438 × 10^7^	4.06 × 10^3^	− 2.116 × 10^4^	6.909 × 10^5^	− 2.536 × 10^4^	− 1.949 × 10^4^	0.229
**ChCl:ox**						
− 2.345 × 10^4^	3.344 × 10^3^	− 1.618 × 10^4^	6.26 × 10^5^	− 2.341 × 10^4^	− 1.603 × 10^4^	0.226
**ChCl:cit**						
4.785 × 10^3^	7.265 × 10^6^	− 1.786 × 10^4^	4.413 × 10^5^	− 2.305 × 10^4^	− 1.522 × 10^4^	0.228

^a^$sd = \left( {\sum\nolimits_{i = 1}^{N} {(w_{1}^{cal} - w_{1}^{\exp } } )^{2} /N} \right)^{0.5}$ where w1 and N represented mass fraction of PPG400 and number of binodal data, respectively.

where $$G_{ij} = \exp ( - \alpha_{ij} \tau_{ij} )$$, $$\alpha_{ij} = \alpha_{ji}$$ and $$\tau_{ij} = \tau_{ji} = 0$$.

The UNIQUAC model, contains two different parts, including combinational and residual. This model is not fully thermodynamically consistent due to its two liquid mixture approach. In this approach the local concentration around one central molecule is assumed to be independent from the local composition around another type of molecule. These parameters always are greater than zero.

12 $$ \ln \gamma_{i} = \ln \gamma_{i}^{c} + \ln \gamma_{i}^{R} $$

13 $$ \ln \gamma_{i}^{c} = \ln \frac{{\varphi_{i} }}{{x_{i} }} + 5q_{i} \ln \frac{{\theta_{i} }}{{\varphi_{i} }} + l_{i} - \frac{{\varphi_{i} }}{{x_{i} }}\sum\limits_{j} {x_{j} l_{j} } $$

14 $$ \ln \gamma_{i}^{R} = q_{i} (1 - \ln (\sum\limits_{j} {\theta_{j} \tau_{ji} ) - \sum\limits_{j} {\frac{{\theta_{j} \tau_{ij} }}{{\sum\limits_{k} {\theta_{k} \tau_{kj} } }}} } $$

15 $$ l_{i} = \frac{z}{2}\left( {r_{i} - q_{i} } \right) - (r_{i} - 1) $$

16 $$ \theta_{i} = \frac{{q_{i} x_{i} }}{{\sum\nolimits_{j} {q_{j} x_{j} } }};\;\;\Phi_{i} = \frac{{r_{i} x_{i} }}{{\sum\nolimits_{j} {r_{j} x_{j} } }} $$

The variables $\theta_{i}$, $\Phi_{i}$, and $\tau_{ji}$ are the volume fraction, area fraction, and interaction parameter between molecule i and j, respectively. The coordination number, Z, the number of molecules surrounding the central molecule, is set to 10. The molecular structure constants of the pure component are q and r and depend on the relative external surface areas and the molecular size (volume), respectively and reported in Table S4.

The fitting parameters and corresponding standard deviations are given in Table 4. The obtained standard deviations (sd) show that both of NRTL and UNIQUAC models can be used to reproduce the binodal data.

The interaction parameters of the NRTL and UNIQUAC models were determined by minimizing the objective function.

17 $$ OF = \sum\limits_{i = 1}^{n} {\left( {\ln \gamma_{i}^{\exp } - \ln \gamma_{i}^{cal} } \right)}^{2} $$

where $\ln \gamma_{i}^{\exp }$ and $\ln \gamma_{i}^{cal}$ are representing the experimental and calculated activity coefficients.

### Correlations of the partition coefficients

For modeling of experimental partition coefficients of 5-HMF molecules in the studied ATPSs, adopted the equation proposed by Diamond-Hsu^49^.

18 $$ \ln K = A \cdot \Delta w(PPG_{400} ) + B \cdot \Delta w(PPG_{400} )^{2} $$

here, *A* and *B* are fitting parameters. The symbol $\Delta w(PPG_{400} )$ is the difference of mass fraction of PPG_400_ in the top and bottom phase:

19 $$ \Delta w(PPG_{400} ) = w(PPG_{400} )_{top} - w(PPG_{400} )_{bottom} $$

In Table 5, the fitting parameters with the corresponding standard deviations, *sd*, are given. According to the the *sd* values, it is concluded that correlation with this used equation is satisfactory for modelling the experimental results Journal standard requires that the first table referenced in the manuscript text should be Table 1, the second, Table 2, etc. However, the original sequence of table citations was out of order. Tables and citations were reordered so that they are cited in consecutive numerical order. Please check if action taken is appropriate. Otherwise, kindly advise us on how to proceed. for the partition coefficients.

**Table 5:** The values of parameters of Diamond-Hsu, Hand and Bachman–Brown equations with the standard deviation of the models, *sd*, for the {PPG400 + ChCl(HBA) + carboxylic acids(HBD) + H_2_O} at 298.15 K and atmospheric pressure (≈ 85 kPa).

DES types	Diamond-Hsu		
	*A*	*10*^*3.*^*B*	*sd*^a^
ChCl:lac	0.0514	− 0.585	0.08
ChCl:ox	0.0279	− 0.261	0.112
ChCl:cit	0.0236	0.0006	0.135

	Hand		
	a_1_	b_1_	R^2^
ChCl:lac	0.5436	0.1971	0.9999
ChCl:ox	0.4449	0.2586	0.9997
ChCl:cit	0.2961	0.2344	0.9998

	Bachman–Brown		
	a_1_	b_1_	R^2^
ChCl:lac	− 3.6905	− 0.1281	0.9998
ChCl:ox	− 3.3644	− 0.1090	0.9996
ChCl:cit	− 3.5128	0.0468	0.9998

### Consistency of tie-line data

The consistency of the experimental tie-line data was always checked by Hand^50^ and Bachman–Brown^42^ equations, which are expressed as:

20 $$ \ln \left( {\frac{{w_{2} }}{{w_{1} }}} \right)^{up} = a_{1} + b_{1} \ln \left( {\frac{{w_{2} }}{{w_{1} }}} \right)^{bot} $$

21 $$ w_{1}^{top} = a_{2} + b_{2} \left( {\frac{{w_{1}^{top} }}{{w_{3}^{bot} }}} \right) $$

The fitting parameters and corresponding linear correlation coefficients, R^2^, are shown in Table 5, which indicates a good consistency for our LLE data. All linear correlation coefficients (R^2^) are close to one; these values indicate of a better goodness of fit for the LLE data of the considered systems.

The AGREE tool is a simple automated and reliable tool with more detailed information about the 12 principles of GAC. The main advantage of AGREE is the clarification of strong and weak sections among the twelve principles of GAC. Besides, its overall score is reliable and informative as regards GAC bases. The AGREE overall greenness score was 0.77 with a green color which indicates the environmental sustainability of the process, (see the Supporting Information). By this technique, the weakest red sections in the AGREE pictogram were criterion 3 (off-line analytical technique).

## Conclusion

Various process intensification techniques have been studied related to the production of 5-HMF, though most are limited to only lab-scale systems. Deep eutectic solvent-based aqueous two phase system was developed to extraction of 5-HMF from aqueous solution.. 5-HMF can be produced to byproducts at high temperatures, therefore, the extraction process is preferably carried out at room temperature. The ATPSs were constructed by acidic DESs (ChCl:lac, ChCl:ox and ChCl:cit) + PPG400 + water while DES-rich, polymer-poor is bottom phase and a polymer-rich, DES-poor is top phase. The experimental LLE data were satisfactorily adjusted by two semi-empirical Zafarani-Moattar et al. and Merchuk equations. Also, the phase behavior of the ATPSs is also satisfactorily described by the NRTL and UNIQUAC models. According to deviations obtained from fitting tie-lines, it was found that performances of these models are good. Finally, the equation proposed by Diamond-Hsu was used for modeling the experimental data of the partition coefficients of 5-HMF and the Hand and Bachman-Brown equations were applied to verify reliability of experimental tie-line compositions. These results indicate the best partitioning with a extraction efficiency of 79% was obtained by the ChCl:lac as DES. These systems have shown the potential to become excellent integrated platforms in which the reaction and separation steps can be carried out sequentially by using their switchable behavior.

## Supplementary Information

Supplementary Information.
