# Supplementary material for: Application of acidic deep eutectic solvents in green extraction of 5-hydroxymethylfurfural
Source: Sci Rep. 2022 Jul 30;12:13113. doi: 10.1038/s41598-022-16823-x (PMC9339007; doi:10.1038/s41598-022-16823-x)
Supplement: Supplementary file 1 — Supplementary Information. [file 41598_2022_16823_MOESM1_ESM.docx]

***Supplementary material***

**Application of acidic deep eutectic solvents in green extraction of 5-hydroxymethylfurfural**

Sabah Karimi, Hemayat Shekaari^*^

Department of Physical Chemistry, Faculty of Chemistry, University of Tabriz, Tabriz, Iran

E-mail address: hemayatt@yahoo.com

Table S1. Values of the parameters and refractive indexes for PPG400 (p), choline chloride (c) and carboxylic acids (l, o and c)

| Material | Constant | Value | C Range (w/w) | ^a^R^2^ |
| --- | --- | --- | --- | --- |
| Choline chloride | *a_c_* | 0.1452 | 0 to 0.08 | 0.9985 |
| PPG400 | *a_p_* | 0.1323 | 0 to 0.1 | 0.9954 |
| Lactic acid | *a_l_* | 0.1435 | 0 to 0.5 | 0.9978 |
| Oxalic acid | *a_o_* | 0.1576 | 0 to 0.5 | 0.9943 |
| Citric acid | *a_c_* | 0.1505 | 0 to 0.7 | 0.9987 |

^a^ where, *R^2^,* represented respective correlation coefficient value of the linear calibration plot of the refractive index against mass fraction of choline chloride, polymer and carboxylic acids at the mass fraction range (*C range*) of each material

**Table S2.** Experimental binodal weight fraction (wt %) data for the systems composed of {ChCl: carboxylic acids (*wNADES* ) + PPG400 ( *wp* ) + H2O} at 298.15 K and atmospheric pressure (≈ 85 kPa).*^a^*

| *W_chcl/lac_* | *W_p_* | *W_chcl/ox_* | *W_p_* | *W_chcl/cit_* | *W_p_* |
| --- | --- | --- | --- | --- | --- |
| 0.0234 | 0.7445 | 0.0247 | 0.7278 | 0.0355 | 0.7307 |
| 0.0306 | 0.7208 | 0.0336 | 0.6827 | 0.0467 | 0.6969 |
| 0.0365 | 0.6911 | 0.0412 | 0.6500 | 0.0546 | 0.6810 |
| 0.0549 | 0.6305 | 0.0623 | 0.5662 | 0.0695 | 0.6477 |
| 0.068 | 0.5869 | 0.0804 | 0.5114 | 0.0833 | 0.6164 |
| 0.0869 | 0.5303 | 0.1055 | 0.4413 | 0.1042 | 0.5783 |
| 0.1023 | 0.4842 | 0.1305 | 0.3725 | 0.124 | 0.5473 |
| 0.1199 | 0.4309 | 0.1508 | 0.3250 | 0.1476 | 0.5097 |
| 0.1345 | 0.3907 | 0.1731 | 0.2827 | 0.1681 | 0.4825 |
| 0.1498 | 0.3498 | 0.1915 | 0.2524 | 0.1916 | 0.4474 |
| 0.1638 | 0.3176 | 0.2128 | 0.2252 | 0.2139 | 0.4169 |
| 0.1779 | 0.2868 | 0.2312 | 0.2048 |  |  |
| 0.1878 | 0.2600 | 0.2482 | 0.1843 |  |  |
| 0.1997 | 0.2373 | 0.2622 | 0.1712 |  |  |
| 0.2133 | 0.2202 | 0.2803 | 0.1573 |  |  |
| 0.2258 | 0.2039 | 0.2947 | 0.1446 |  |  |
| 0.2373 | 0.1907 | 0.3098 | 0.1339 |  |  |
| 0.249 | 0.1767 | 0.3251 | 0.1250 |  |  |
| 0.262 | 0.1676 | 0.3402 | 0.1161 |  |  |
| 0.2669 | 0.1534 |  | 0.7278 |  |  |
| 0.2801 | 0.1458 |  | 0.6827 |  |  |

*^a^* The standard uncertainties *σ* for mass fraction, temperature and pressure are: *σ* (*wi*) = 0.002; *σ* (*T*) = 0.05K and *σ* (*p*) = 0.05 kPa, respectively

**Table S3.** Experimental tie-line data of {PPG400 + ChCl (HBA) + carboxylic acids (HBD) + H2O} systems at 298.15 K and atmospheric pressure (≈ 85 kPa).*^a^*

| Overal composition / wt% | | |  | PPG-rich phase composition / wt% | | |  | ChCl-rich phase composition / wt% | | |  | TLL |
| --- | --- | --- | --- | --- | --- | --- | --- | --- | --- | --- | --- | --- |
| [HBA:HBD] | | [PPG] |  | [HBA] | [HBD] | [PPG] |  | [HBA] | [HBD] | [PPG] |  |  |
| ChCl:lac | | | | | | | | | | | | |
| 0.142 | 47.692 | |  | 0.017 | 0.003 | 0.80 |  | 0.195 | 0.040 | 0.2 |  | 62.69 |
| 0.171 | 47.584 | |  | 0.016 | 0.003 | 0.85 |  | 0.235 | 0.05 | 0.16 |  | 72.54 |
| 0.200 | 47.254 | |  | 0.017 | 0.0033 | 0.90 |  | 0.27 | 0.059 | 0.13 |  | 81.24 |
| 0.229 | 47.842 | |  | 0.016 | 0.0035 | 0.93 |  | 0.329 | 0.063 | 0.13 |  | 86.11 |
| 0.258 | 47.472 | |  | 0.017 | 0.0037 | 0.95 |  | 0.37 | 0.063 | 0.12 |  | 90.39 |
| ChCl:ox | | | | | | | | | | | | |
| 0.165 | 0.3897 | |  | 0.01 | 0.0150 | 0.77 |  | 0.150 | 0.101 | 0.17 |  | 62.17 |
| 0.194 | 0.3854 | |  | 0.012 | 0.0153 | 0.82 |  | 0.172 | 0.117 | 0.15 |  | 69.60 |
| 0.223 | 0.3895 | |  | 0.011 | 0.0153 | 0.87 |  | 0.197 | 0.131 | 0.12 |  | 78.10 |
| 0.252 | 0.3805 | |  | 0.013 | 0.0155 | 0.92 |  | 0.215 | 0.147 | 0.11 |  | 84.49 |
| 0.281 | 0.3854 | |  | 0.012 | 0.0154 | 0.95 |  | 0.248 | 0.194 | 0.09 |  | 90.93 |
| ChCl:cit | | | | | | | | | | | | |
| 0.129 | 57.05 | |  | 0.0130 | 0.010 | 0.83 |  | 0.070 | 0.081 | 0.50 |  | 34.23 |
| 0.145 | 57.15 | |  | 0.0140 | 0.011 | 0.86 |  | 0.102 | 0.090 | 0.45 |  | 42.67 |
| 0.161 | 57.54 | |  | 0.0143 | 0.012 | 0.89 |  | 0.107 | 0.111 | 0.42 |  | 48.92 |
| 0.177 | 57.46 | |  | 0.0141 | 0.012 | 0.90 |  | 0.115 | 0.127 | 0.40 |  | 52.29 |
| 0.193 | 57.18 | |  | 0.0142 | 0.013 | 0.94 |  | 0.148 | 0.134 | 0.38 |  | 58.83 |

^a^ The standard uncertainty for mass percent of each component is 0.8.

**Table S4**. The UNIQUAC *r* and *q* parameters for the used materials and DESs.

| *q* | *r* | Materials/DESs |
| --- | --- | --- |
| 5.100 | 6.128 | Choline chloride |
| 4.476 | 5.274 | Lactic acid |
| 13.064 | 14.638 | Oxalic acid |
| 4.808 | 5.958 | Citric acid |
| 4.954 | 3.795 | PPG400 |
| 1.399 | 0.920 | Water |
| 4.788 | 5.701 | ChCl:lac |
| 9.082 | 10.383 | ChCl:ox |
| 4.954 | 6.043 | ChCl:cit |

**
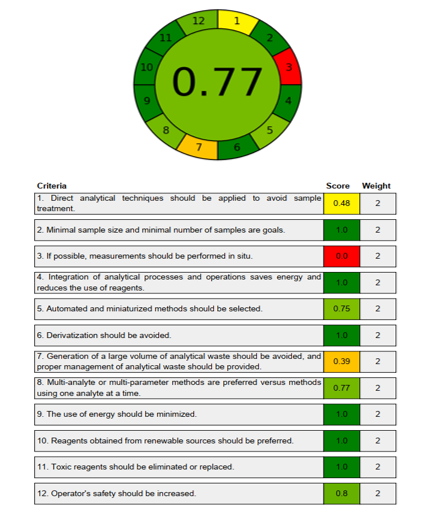
**

**Fig S1.** AGREE tools for assessment of greenness values of protocol.
